# Bougies as an aid for endotracheal intubation with the Airway Scope: bench and manikin comparison studies

**DOI:** 10.1186/s12871-017-0424-1

**Published:** 2017-10-02

**Authors:** Ichiro Takenaka, Kazuyoshi Aoyama, Tamao Iwagaki, Yukari Takenaka

**Affiliations:** 1 Department of Anesthesia, Kyushu Rosai Hospital, 1-1 Sonekita, Kokuraminami, Kitakyushu, 800-0296 Japan; 2 Department of Anesthesia, Kitakyushu General Hospital, 1-1 Higashijono, Kokurakita, Kitakyushu, 802-8517 Japan

**Keywords:** Endotracheal intubation Videolaryngoscopy Bougie

## Abstract

**Background:**

When encountering a difficult airway with an Airway Scope (AWS) a bougie can be inserted into the endotracheal tube in the AWS channel. The angulated tip of the bougie can be guided toward the glottis by rotating it. We tested the ease of rotating bougies (Venn reusable, Boussignac, Portex single-use, and Frova) in an endotracheal tube when placed in the AWS channel.

**Methods:**

Bench study: Seven anesthesiologists inserted each of the four types of bougies into a 7.0 mm endotracheal tube in an AWS channel and rotated the bougie end (side of bougie operated by hand) clockwise or counterclockwise to an angle of 0°-180° in 45° increments. The rotation angle of the bougie tip (tracheal side) was measured for each bougie and the degree of force required to rotate them was examined. Manikin study: Using the same four bougies, the same seven anesthesiologists attempted to intubate a manikin that simulated a difficult airway. Success rate and time required for successful intubation were compared between the four bougies.

**Results:**

Bench study: The difference in the rotation angle between the bougie tip and end was significantly larger with Portex single-use and Frova bougies than with Venn reusable and Boussignac bougies (*P* < 0.01). The rotation angles of the tips of Venn reusable, Boussignac, Portex single-use, and Frova bougies were 145°/123° (clockwise / counterclockwise), 92°/108°, 46°/56°, and 39°/51°, respectively, when their ends were rotated to an angle of 180°. Venn reusable and Boussignac bougies could be rotated in the endotracheal tube by clinically acceptable rotational force. Manikin study: Times to intubation with Venn reusable [25 (SD, 5) s] and Boussignac bougies [35 (6) s] were significantly shorter than with Portex single-use [61 (17) s] and Frova bougies [69 (22) s] (*P* < 0.01). There were no significant differences in success rate between the four bougies.

**Conclusions:**

Venn reusable and Boussignac bougies are a useful aid for intubation with an AWS. Portex single-use and Frova bougies seem to be less suitable for this technique. Different bougies may be of varying utility when used with an AWS or airway device with an endotracheal tube channel.

## Background

The Airway Scope (AWS, HOYA-Pentax, Tokyo, Japan) belongs to the family of channeled videolaryngoscopes with a built-in monitor, like the Airtraq® (Prodol Meditec S.A., Vizcaya, Spain) or the King Vision® videolaryngoscope (Ambu Inc., Ballerup, Denmark). The blade is designed to match the anatomy of the upper airway and provides an excellent view of the glottis, even in patients with difficult airways [1, 2]. During attempts at endotracheal intubation with an AWS, the curvature of an endotracheal tube and an endotracheal tube guiding channel of the AWS blade determines the direction in which the tube advances. This direction is thus fixed, which is displayed on the AWS’ monitor screen as a target symbol. The target symbol is aligned with the glottis to achieve insertion of the tube into the trachea [1, 3]. Failure to align the target symbol with the glottis prevents insertion of an endotracheal tube into the trachea, making intubation with an AWS difficult. Moreover, a direct lift of the epiglottis with the blade tip is necessary for successful intubation with an AWS. Failure to achieve this can result in the advancing tube impinging on the epiglottis [4–6].

Because a thin angulated tip can easily be manipulated in a desired direction, a small bougie with an angulated tip can facilitate precise insertion, even into a small target [6–9]. Such a bougie can be passed through the narrow curved lumen of the endotracheal tube that is placed into the AWS channel and can be advanced into the glottis and trachea, even when the target symbol cannot be aligned with the glottis or the epiglottis cannot be lifted directly with the tip of the AWS blade [4–6]. When using a bougie as an aid to intubation with an AWS, the laryngoscopist often attempts to guide its angulated tip toward the glottis by rotating its end that is closest to the airway adaptor of the endotracheal tube. However, rotation of the bougie within the tube may result in twisting of its shaft, preventing full transmission of the rotational force applied at its end to the angulated tip. To the best of our knowledge, no published studies have examined which bougies can be freely and easily rotated in the lumen of the endotracheal tube set in the curved blade of an AWS. We therefore examined the ease or difficulty of rotating four commercially available bougies within the endotracheal tube inserted into an AWS channel and the efficacy of these bougies in a difficult intubation simulation manikin.

## Methods

### Bench study

**Fig. 1:**
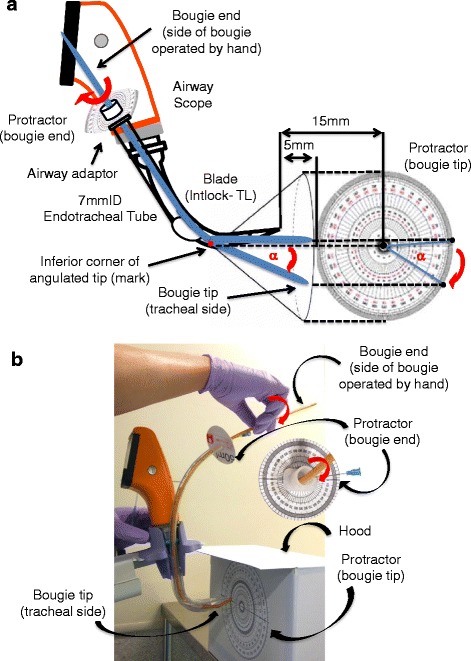
Experimental protocol for the bench study. According to the instruction manual [3], a 7.0 mm internal diameter standard endotracheal tube is inserted into the guiding channel of an Airway Scope blade, the tip of which is set 15 mm from the circular protractor that stands vertically on a table (**a**). The anesthesiologist marks the inferior corner of the angulated tip of the bougie then, with the angulated tip directed towards 12 o’clock, inserts the bougie into the tube, locates the tip 5 mm beyond the blade tip, and aligns the mark with the center of the protractor on the monitor screen of the AWS (**a**). The power switch of the Airway Scope is turned off. The anesthesiologist then rotates the bougie end (side of bougie operated by hand) clockwise or counterclockwise to an angle of 0°–180° in 45° increments while looking at the protractor that is set on the airway adaptor of the endotracheal tube (**b**). The bougie tip (tracheal side) is not visible because of the hood (**b**). The angle of the bougie tip created by rotating its end is measured. α: rotation angle of the bougie tip

Seven anesthesiologists with at least 5 years experience participated in this study as follows (Fig. 1). They examined four commercially available bougies: Venn reusable tracheal tube introducers (Smiths Medical, Keene, NH, USA), Boussignac bougies (Vygon, Ecouen, France), Portex single-use bougies (Smiths Medical, Keene, NH, USA), and Frova intubating catheters (Cook Medical, Bjaeverskov, Denmark) (Table 1). The order of testing of the bougies and the direction of rotation were randomly assigned by a sealed envelope technique. A 7.0 mm internal diameter standard endotracheal tube (Smiths Medical, Tokyo, Japan) was inserted into the endotracheal tube guiding channel of an AWS blade (Intlock-TL, HOYA-Pentax, Tokyo, Japan) and the tip of the endotracheal tube was located beside the scope window of the AWS blade [3]. The tip of the AWS blade was set 15 mm from the circular protractor that was standing vertically on a table. The inferior corner of the angulated tip of each bougie was marked. After the bougie had been thoroughly lubricated with 8% lidocaine spray, it was inserted into the endotracheal tube with its angulated tip, which was located 5 mm beyond the blade tip, being directed towards 12 o’clock. The mark indicating the inferior corner of the angulated tip of the bougie was then aligned with the center of the circular protractor on the monitor screen of the AWS. The power switch of the AWS was turned off and the bougie tip (tracheal side) was concealed with the hood. The bougie end (side of bougie operated by hand) was then rotated clockwise or counterclockwise to an angle of 0° –180° in 45° increments as measured by another circular protractor that was set on the airway adaptor of the endotracheal tube. The furthest the bougie tip reached after maximal rotation was marked on the circular protractor. The degree of force required to rotate the bougie was subjectively classified as easy, moderate, difficult, and impossible for each bougie. The bougie was then exchanged in accordance with a predetermined sequence and the rotation angles of its tip were measured in the same fashion. Assistants, who were blinded to the purpose of the study and the type of bougie, calculated the rotation angles on the circular protractor.

**Table 1:** Relevant characteristics of the four commercially available bougies tested

Bougie	Length (cm)	Diameter (Fr)	Angulated tip	Construction material	Product
Venn reusable	60	15	2.5 cm / 35°	inner: polyester outer: resin	Smiths Medical
Boussignac	70	15	2.5 cm / 40°	polyether block amide	Vygon
Portex single-use	70	15	2 cm / 35°	polyvinyl chloride	Smiths Medical
Frova	70	14	3 cm / 30°	polyethylene	Cook Medical

### Manikin study

A manikin was used to simulate a difficult airway. In this manikin the epiglottis could not be lifted directly with the AWS blade [6]. The same seven anesthesiologists attempted to intubate the manikin’s trachea using the same four bougies. An AirSim manikin (TruCcorp, Belfast, Northern Ireland) was utilized, its tongue being inflated with 75 ml of air, which provided the Cormack and Lehane grade 3 view with a Macintosh laryngoscope. The order of testing of the bougies was randomly assigned by a sealed envelope technique. A 7.0 mm internal diameter standard endotracheal tube was inserted into the AWS channel. After the bougie had been thoroughly lubricated with 8% lidocaine spray, it was inserted into the tube with its angulated tip directed towards 12 o’clock. The manikin was placed supine without a pillow on a flat table. Next, the anesthesiologist inserted an AWS blade into its mouth until its tip was positioned in the vallecula. After the glottis had been fully exposed, the laryngoscopic view achieved was assessed using Cormack and Lehane’ classification. The anesthesiologist then attempted to guide the bougie tip toward the glottis by rotation, inserted the bougie into the trachea, and advanced the endotracheal tube over it. An assistant measured time to intubation, which was defined as the time from insertion of the AWS blade between the teeth to the endotracheal tube cuff passing through the vocal cords. Passage of the cuff was confirmed on the monitor screen of the AWS. The attempt was deemed a failure if the intubation attempt took longer than 120 s or the endotracheal tube was inserted into the esophagus. The bougies were exchanged in accordance with a predetermined sequence and the anesthesiologists were attempting intubation with each of them in the same fashion. The laryngoscopic grade, time required for successful intubation, and success or failure of intubation were recorded.

### Statistical analysis

To evaluate the performance of each bougie as an aid to endotracheal intubation with an AWS, the differences in rotation angle between its end and tip, which were the primary endpoint, were calculated. A preliminary study had established that the difference in rotation angle was 129° with a SD of 13° when a Portex single-use bougie end was rotated clockwise to an angle of 180° (unpublished data presented at the 63th Annual Meeting of the Japanese Society of the Anesthesiologists, Fukuoka, 26 May, 2016). With a 20% absolute change defined as clinically important change [10], it was calculated that a minimum sample size of six was required to detect such a change with α = 0.05 and β = 0.2. Significant differences were tested for by one-way ANOVA with a Bonferroni post hoc correction for multiple comparisons. Rotation of the bougie by an easy or moderate degree of force was considered clinically acceptable and Fisher’s exact probability test was used for comparison. Differences in the Cormack-Lehane laryngoscopic grade and success rate were analyzed by Kruskal-Wallis test and the chi-square test, respectively. A *p* value <0.05 was considered to denote significance. Statistical analysis was carried out using StatView 5.0 (SAS Institute, Cary, NC, USA).

## Results

### Bench study

#### Difference in rotation angle between the bougie tip and end (Fig. 2)

**Fig. 2:**
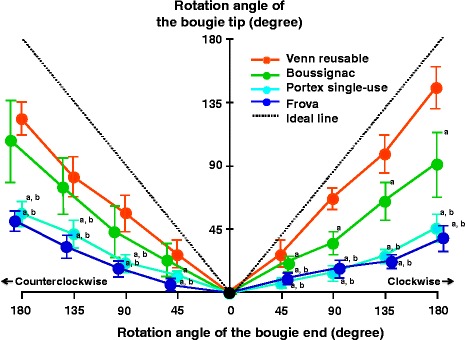
Mean (SD) of rotation angle of the tip of each bougie created by rotating its end. Ideal line (*dotted line*) denotes complete transmission of the rotational force at the bougie end along the shaft to its tip. The difference between the ideal line and each colored line is that in rotation angle between its end and tip. ^a^ *P* < 0.01 vs Venn reusable. ^b^ *P* < 0.01 vs Boussignac bougie

Figure 2 shows the rotation angles of the tip of each bougie created by rotating its end. The differences in the rotation angle between the bougie tip and end were significantly larger with Portex single-use and Frova bougies than with Venn reusable and Boussignac bougies (*P* < 0.01). The rotation angles of the tips of Venn reusable, Boussignac, Portex single-use, and Frova bougies were 145°/123° (clockwise / counterclockwise), 92°/108°, 46°/56°, and 39°/51°, respectively, when their ends were rotated clockwise or counterclockwise to an angle of 180°. Venn reusable and Boussignac bougies could be rotated in an endotracheal tube placed into an AWS channel, the former achieving superior values. At all angles that we measured, the rotation achieved at the tips of Portex single-use and Frova bougies was less than a one-third of that applied at their ends.

#### Degree of force required to rotate the bougie (Table 2)

All seven anesthesiologists could rotate Venn reusable and Boussignac bougies by applying easy or moderate rotational force to their ends; however, rotation of Portex single-use and Frova bougies was difficult or impossible. Venn reusable and Boussignac bougies could be rotated easier than Portex single-use and Frova bougies (*P* < 0.01).

**Table 2:** Degree of force required to rotate the bougie tips as classified by each anesthesiologist

Degree of force for rotation	Bougie			
	Venn reusable	Boussignac	Portex single-use^a, b^	Frova^a, b^
Easy	7	5	0	0
Moderate	0	2	0	0
Difficult	0	0	6	5
Impossible	0	0	1	2

Easy or moderate is considered clinically acceptable

^a^ *P* < 0.01 vs Venn reusable

^b^ *P* < 0.01 vs Boussignac

### Manikin study

#### Time required for successful intubation (Table 3)

Mean times required for successful intubation with Venn reusable, Boussignac, Portex single-use, and Frova bougies were 25 (SD, 5) s, 35 (6) s, 61 (17) s, and 69 (22) s, respectively. Intubation times were significantly shorter with Venn reusable and Boussignac bougies than with Portex single-use and Frova bougies (P < 0.01) despite no significant differences in Cormack-Lehane laryngoscopic grade between the four bougies.

**Table 3:** Laryngoscopic view, success rate, and time required for successful intubation with an Airway Scope and bougie in a manikin

	Bougie			
	Venn reusable	Boussignac	Portex single-use	Frova
Laryngoscopic view (grade1/2/3)	6/1/0	7/0/0	7/0/0	7/0/0
Success rate	7/7	7/7	5/7	4/7
Time required for successful intubation (second)	25(5)	35(6)	61(17)^a,b^	69(22)^a,b^

Values for time to intubation are mean (SD)

^a^ *P* < 0.01 vs Venn reusable

^b^ *P* < 0.01 vs Boussignac

#### Number of successful intubations (Table 3)

All seven anesthesiologists achieved endotracheal intubation with Venn reusable and Boussignac bougies but 2 and 3 of them failed to achieve with Portex single-use and Frova bougies, respectively. There was no significant difference in success rate between the four bougies.

## Discussion

Bench study demonstrated that Venn reusable and Boussignac bougies could be rotated without difficulty in the lumens of endotracheal tubes placed into the channel of an AWS. However, the rotation achieved at the tips of Portex single-use and Frova bougies was less than a one-third of that applied at their ends because their shafts twisted, preventing full transmission of the rotational force applied at their ends to their tips.

Laryngoscopists must rotate the bougies with one hand only because they have to hold the AWS in the other hand. Thus, bougies that require application of excessive force to achieve free rotation of the bougie tip in the endotracheal tube are not useful aids to intubation with an AWS. Of the four bougies that we studied, only Venn reusable and Boussignac bougies could be easily rotated in the lumens of the endotracheal tubes by applying an easy or moderate degree of rotational force, which we considered clinically acceptable, to their ends; thus these bougies were clinically useful aids to intubation with an AWS. In contrast, rotation of the ends of Portex single-use and Frova bougies to 180°, which required excessive force, achieved less than 56° rotation of their tips.

In the scenario of difficult intubation with an AWS that we simulated, four steps were required to achieve insertion of a bougie into the trachea: 1) directing the angulated tip towards 6 o’clock by rotation; 2) positioning it behind the epiglottis; 3) changing the direction of the tip from 6 o’clock to 12 o’clock; and 4) advancing the bougie through the vocal cords while keeping close to the laryngeal surface of the epiglottis. The anesthesiologists were able to insert Venn reusable and Boussignac bougies into the trachea without difficulty because their tips rotated easily. In contrast, even when the anesthesiologists used considerable rotational force to rotate the ends of Portex single-use and Frova bougies, their tips did not rotate freely and could not be guided in the desired direction. Thus, it was often difficult for the anesthesiologists to place the bougies’ tips behind the epiglottis, which prolonged time required for the intubation attempt and made achievement of endotracheal intubation difficult. These findings supported the results of the bench study. We therefore recommend using Venn reusable or Boussignac bougie, particularly in patients whose trachea are difficult to intubate with an AWS.

Even when an excellent view of the glottis has been achieved, it is sometimes difficult to insert an endotracheal tube along the anatomical curve-shaped blade of a videolaryngoscope across the vocal cords [4–6, 11–15]. This is a common difficulty in endotracheal intubation using videolaryngoscopes [11]. Previous studies have suggested that bougies can effectively address this problem [1, 4–6, 11–15]. For example, bougies are reportedly useful for assisting intubation with an Airtraq [14, 15]. We believe that our findings are applicable to addressing difficult intubation using the family of channeled videolaryngoscopes with anatomically-shaped blade and a guiding channel that is designed to direct the endotracheal tube toward the glottis.

Several factors contribute to rotating a bougie in the lumen of an endotracheal tube; these comprise the construction material, size, curvature, structure of the inner surface of the endotracheal tube, lubrication, and the construction material of the bougie. In this study, the endotracheal tube and lubricant were identical in all tests. We believe that the construction material of the bougie may influence its rotation in the endotracheal tube. The four bougies tested were constructed with different polymers (Table 1), which had different torsion strength properties. The polymers of Portex single-use and Frova bougies might tend to twist against the friction that occurred during rotation in the narrow curved lumen of the tube, compared with those of Venn reusable and Boussignac bougies. We consider that the difference in their properties is a possible explanation for difficult rotation. Further studies are needed.

Airway trauma can occur during intubation with a bougie as a result of aggressive placement of the bougie or railroading the endotracheal tube over it. However, this complication has been reported even when the bougie has been advanced without resistance and the tube easily railroaded over it [16]. Moreover, there have been some cases of displacement of the epiglottis and its prolapse into the trachea beside the endotracheal tube during attempts of intubation with an AWS [17]. Anesthesiologists should be aware of these possible complications of intubation with a bougie and/or an AWS, and be mindful to manipulate the bougie and endotracheal tube gently and carefully during intubation attempts. If an injury of the larynx and trachea is suspected, it should be identified directly or with a fiberscope.

### Study limitations

Our experimental design has some potential limitations. It was impossible to blind the anesthesiologists to the bougie being used, potentially biasing measurement of the rotation angle of the bougie tip. In addition, the seven anesthesiologists who did the bench study were the same as the ones who did the manikin study, which might cause a potential for bias in the results of the manikin study.

Regarding measuring the angle of rotation of the bougies’ tips, we postulated that they rotated around the corner of the angulated tip that had been marked. However, when a bougie end was rotated, its tip did not always draw a perfect circle because the center sometimes moved, potentially introducing bias in measuring the angle of rotation. When using a bougie as an aid to intubation with an AWS, precise rotation of the bougie tip may be unnecessary because it is visible on the monitor screen. Ease of manipulation of the tip in a desired direction should be more important than precise rotation. In the bench study, it was clearly easier to rotate Venn reusable and Boussignac bougies than Portex single-use and Frova bougies. Moreover, in the manikin study, in which the epiglottis could not be directly lifted with the tip of the AWS blade, the 180° rotation of the bougie tip that was required for successful intubation was often difficult to achieve with Portex single-use bougies and Frova intubating catheters. Thus, we believe that these biases were of minor importance.

We used an AirSim manikin in this study. A preliminary assessment of the ability of various commercially available manikins to simulate a scenario of difficult intubation with an AWS, which differs from that with a conventional laryngoscope, resulted in us choosing an AirSim manikin as the most suitable for our purposes. Notably, this was a bench and manikin study aimed at comparing four bougies, the findings may not accurately indicate how each bougie performs in humans.

Because our study was designed for a scenario of difficult intubation with an AWS, we used a relatively small size endotracheal tube. Further studies using larger endotracheal tubes are therefore needed.

Bougies are designed as aids to endotracheal intubation with a conventional laryngoscope rather than devices for rotating in the narrow curved lumens of the endotracheal tubes [6–8]. Thus, the results of this study do not reflect the performance of these bougies when used for their original purposes.

## Conclusions

We have demonstrated by both bench and manikin studies that the four bougies that we studied are not equivalent in terms of their rotation in the lumens of endotracheal tubes placed into the channel of an AWS. Of the four bougies investigated, we found that Venn reusable tracheal tube introducers and Boussignac bougies are useful aids to intubation with an AWS. Portex single-use bougies and Frova intubating catheters seem to be less suitable for this technique. Different bougies may be of varying utility when used with an AWS or airway device with an endotracheal tube channel. We recommend confirming that the bougie to be used does rotate easily within the endotracheal tube placed into the channel of an AWS before using it.

## Abbreviations

ANOVA: Analysis of variance
AWS: Airway Scope
s: second
SD: Standard deviation
